# Association of the Hemoglobin–Albumin–Lymphocyte–Platelet (HALP) Score with Diabetic Retinopathy Severity: A Case–Control Comparison with Non-Diabetic Ophthalmology Patients

**DOI:** 10.3390/jcm15156112

**Published:** 2026-08-06

**Authors:** Nurcan Gürsoy, Ersan Gürsoy

**Affiliations:** 1 Department of Ophthalmology, Of State Hospital, Trabzon 61830, Türkiye; 2 Department of Family Medicine, Trabzon University Faculty of Medicine, Trabzon 61030, Türkiye; ersangursoy@trabzon.edu.tr

**Keywords:** diabetic retinopathy, biomarkers, disease progression, inflammation, nutritional status

## Abstract

**Background/Objectives:** The hemoglobin–albumin–lymphocyte–platelet (HALP) score combines routine laboratory measures into an index of systemic immune–nutritional status. Its relationship with graded diabetic retinopathy (DR) severity is not fully established. We compared HALP between patients with DR and ophthalmology controls without documented diabetes and examined its association with retinopathy stage and proliferative disease. **Methods:** This prospective single-center case–control study enrolled 209 consecutive adults: 105 patients with DR and 104 controls. DR was recorded across eight ordered stages and additionally classified as non-proliferative DR (NPDR) or proliferative DR (PDR). Between-group tests, Spearman correlation, logistic regression, and receiver operating characteristic analysis were used. **Results:** HALP was lower in the DR group than in controls (47.47 [26.44] vs. 52.67 [31.83], *p* = 0.048) and in PDR than in NPDR (45.98 [17.62] vs. 52.90 [42.49], *p* = 0.008). HALP declined modestly with increasing stage (rho = −0.260, *p* = 0.007). In an exploratory age- and sex-adjusted model, each 10-unit increase in HALP was associated with lower odds of case-group membership (OR = 0.850, 95% CI: 0.750–0.963; *p* = 0.011). Within the DR cohort, HALP was not significantly associated with PDR after adjustment for age, sex, and HbA1c (OR = 0.809, 95% CI: 0.640–1.023; *p* = 0.077). Discrimination between cases and controls was limited (AUC = 0.579); a threshold of ≤52.09 provided 62.9% sensitivity and 53.8% specificity. **Conclusions:** Lower HALP values accompanied DR case status and greater retinopathy severity, but the adjusted association with PDR was not significant. Because diabetes status differed between groups, the case–control finding may reflect systemic effects of diabetes as well as retinal involvement.

## 1. Introduction

Diabetic retinopathy (DR) is one of the most consequential microvascular complications of diabetes mellitus and remains a major cause of preventable visual impairment among working-age adults [1]. Among individuals with diabetes, its global prevalence has been estimated at 22.3%, corresponding to approximately 103 million affected adults in 2020, and this number is projected to rise to 160.5 million by 2045 [2]. The number of individuals with vision-threatening DR is likewise expected to rise from 28.5 million to 44.8 million over the same period [2]. This expanding burden highlights the need for readily accessible systemic indicators that may complement ophthalmologic evaluation and contribute to the clinical characterization of patients with more advanced retinal involvement [2,3].

The development and progression of DR reflect a complex interaction of chronic hyperglycemia, retinal microvascular injury, oxidative stress, inflammation, hypoxia, and angiogenic activation [4,5,6]. These processes are not confined to the retina; systemic inflammatory and nutritional disturbances may also influence endothelial integrity, tissue oxygenation, and the response of retinal tissue to sustained metabolic stress [7,8]. Accordingly, routinely available hematologic indices have gained attention as low-cost adjunctive markers in DR. Several studies have demonstrated associations of the neutrophil-to-lymphocyte ratio, platelet-to-lymphocyte ratio, and systemic immune–inflammation index with the presence and stage of DR [9,10,11]. More recent evidence concerning albumin-based composite indices further supports the concept that combining inflammatory and nutritional information may provide a broader representation of systemic status than isolated blood-cell parameters alone [12].

The hemoglobin–albumin–lymphocyte–platelet (HALP) score integrates oxygen-carrying capacity, nutritional–inflammatory status, immune competence, and platelet-related vascular activity into a single composite index [13]. Its individual components provide complementary biological information: hemoglobin reflects systemic oxygen-carrying capacity; albumin is influenced by nutritional reserve, inflammation, and antioxidant capacity; lymphocyte count provides information regarding immune competence; and platelet count may reflect vascular and thrombotic activity [13]. Lower HALP values may reflect a combined state of reduced oxygen-carrying capacity, impaired nutritional–inflammatory reserve, and systemic immune dysregulation [13]. This multidimensional structure provides a biological rationale for investigating HALP in a retinal disorder characterized by inflammation, ischemia, endothelial injury, and pathological neovascularization.

Evidence linking HALP to diabetic retinopathy remains limited but has become increasingly consistent across different diabetic populations. A nationally representative US analysis demonstrated a nonlinear, L-shaped inverse association between HALP and DR [14]. A clinical study involving patients with type 2 diabetes subsequently found that lower HALP values were independently associated with DR and showed modest discriminatory performance [15]. Another clinical investigation reported progressively lower HALP scores across patients without retinopathy, those with diabetic retinopathy, and those with proliferative disease, suggesting that HALP may also vary with disease advancement [16]. More recently, a retrospective study of 193 patients with type 1 diabetes similarly found significantly lower HALP scores in participants with DR than in those without retinal involvement [17]. Collectively, these findings support an inverse relationship between HALP and DR across different diabetic populations. However, evidence remains scarce regarding whether HALP is independently associated with proliferative disease after adjustment for relevant clinical and metabolic confounders.

Although the available studies generally support an inverse relationship between HALP and diabetic retinopathy, the evidence base remains limited and methodologically heterogeneous in terms of diabetes type, comparator definitions, and analytical approach [14,15,16,17]. Previous investigations have primarily examined the presence of DR [14,15,17], whereas disease severity has generally been evaluated using broad categorical comparisons, such as groups without DR, with DR, and with PDR [16]. More detailed assessment across ordered retinopathy stages and multivariable evaluation of the relationship between HALP and PDR have received comparatively limited attention. Accordingly, the primary aim of this study was to compare HALP scores between patients with DR and ophthalmology controls without a documented diagnosis of diabetes. The secondary aims were to examine the relationship between HALP and eight ordered retinopathy stages, compare HALP scores between patients with NPDR and PDR, and determine whether HALP was associated with PDR after adjustment for age, sex, and HbA1c.

## 2. Materials and Methods

### 2.1. Study Design and Setting

Adult participants were prospectively enrolled at the Ophthalmology Clinic of Erzincan Binali Yıldırım University Mengücek Gazi Training and Research Hospital from October 2025 through April 2026. Eligibility screening was performed consecutively among individuals presenting to the outpatient clinic. Patients with established diabetes and clinically confirmed DR constituted the case cohort. The comparison cohort comprised routine ophthalmology patients with no known or recorded diagnosis of diabetes. To assess sample adequacy for the primary comparison of HALP scores between the DR and control groups, a calculation based on a two-sided independent-samples comparison was performed using G*Power version 3.1.9.7 (Heinrich Heine University Düsseldorf, Düsseldorf, Germany). A standardized effect size of Cohen’s d = 0.43, based on the between-group effect reported by Wang et al. [15], was used. Assuming a two-sided significance level of 0.05, 80% statistical power, and equal group allocation (1:1), the minimum required sample size was 172 participants, corresponding to 86 participants per group. The final sample of 209 participants exceeded this requirement. This calculation applied to the primary between-group comparison and was not intended to establish the power of the adjusted PDR subgroup analysis.

### 2.2. Participant Selection and Eligibility Criteria

The study included 209 participants, comprising 105 patients with diabetic retinopathy and 104 control participants without a known history or documented diagnosis of diabetes mellitus. Participants in both groups were recruited consecutively from eligible patients attending the ophthalmology outpatient clinic.

Eligible participants in the diabetic retinopathy group were adults with a documented diagnosis of diabetes mellitus, clinically diagnosed diabetic retinopathy, a completed bilateral ophthalmologic examination, and available laboratory data sufficient to calculate the HALP score. Eligible control participants were consecutively recruited adults who attended the ophthalmology outpatient clinic for routine evaluation, had no known history or documented diagnosis of diabetes mellitus, and had the laboratory data required for HALP calculation.

Patients were excluded if they had an acute or chronic infectious disease, an active systemic inflammatory or autoimmune disorder, active malignancy, clinically significant renal or hepatic disease, anemia, hypoalbuminemia, or another condition likely to substantially affect the components of the HALP score. Anemia and hypoalbuminemia were defined according to the reference ranges of the institutional laboratory. Participants receiving systemic corticosteroids or other immunosuppressive treatments were also excluded. Control participants with age-related macular degeneration, retinal vein or artery occlusion, retinal dystrophy, or any other clinically significant retinal disease were not included. In the diabetic retinopathy group, patients with a history of retinal laser photocoagulation, intravitreal injection, or vitreoretinal surgery were excluded.

### 2.3. Clinical and Laboratory Data

Age, sex, hemoglobin concentration, platelet count, lymphocyte count, serum albumin, high-density lipoprotein cholesterol, and the calculated HALP score were recorded for both groups. Serum glucose and HbA1c values were additionally available for patients with diabetic retinopathy. Hemoglobin concentration, lymphocyte count, and platelet count were measured using a Sysmex XN-2000 automated hematology analyzer (Sysmex Corporation, Kobe, Japan). Serum albumin, glucose, and HDL cholesterol levels were measured using a Beckman Coulter AU2700 chemistry analyzer (Beckman Coulter, Inc., Brea, CA, USA). HbA1c was measured by high-performance liquid chromatography using a Tosoh HLC-723 G8 automated glycohemoglobin analyzer (Tosoh Corporation, Tokyo, Japan). Glucose and HbA1c were not measured in the control group because the participants had no documented diagnosis of diabetes, these tests were not clinically indicated as part of their routine ophthalmologic care, and no study-specific biochemical testing was performed solely for research purposes.

### 2.4. Ophthalmologic Evaluation and Retinopathy Grading

Both eyes were evaluated after pharmacological pupil dilation by the same experienced ophthalmologist using a Nidek slit-lamp biomicroscope (Nidek Co., Ltd., Gamagori, Aichi, Japan) and a 90 D non-contact fundus lens (Volk Optical Inc., Mentor, OH, USA). The ophthalmologist was not formally blinded to the individual laboratory results; however, the HALP score was calculated only after completion of the retinopathy grading and was therefore unavailable at the time of grading. Diabetic retinopathy severity was classified using an ETDRS-derived clinical grading scheme [18]. The eight ordered categories were: (1) very mild NPDR, (2) mild NPDR, (3) moderate NPDR, (4) severe NPDR, (5) very severe NPDR, (6) early PDR, (7) high-risk PDR, and (8) advanced PDR. Severe NPDR was identified according to the 4-2-1 rule, whereas PDR was defined by the presence of retinal or optic-disc neovascularization, preretinal hemorrhage, and/or vitreous hemorrhage. Both eyes were graded separately, and the eye with the more advanced stage was used for participant-level classification. For subgroup analyses, stages 1–5 were categorized as NPDR and stages 6–8 as PDR. Diabetic macular edema was assessed during the ophthalmologic examination but was outside the prespecified study outcomes and was not included in the analyses.

### 2.5. HALP Score Calculation

The hemoglobin–albumin–lymphocyte–platelet (HALP) score was calculated using the following formula:

$$HALP\;score=\frac{Hemoglobin\;(g/L)\times Albumin\;(g/L)\times Lymphocyte\;count\;(10^{9}/L)}{Platelet\;count\;(10^{9}/L)}$$

### 2.6. Ethical Considerations

The study was approved by the Erzincan Binali Yıldırım University Non-Interventional Clinical Research Ethics Committee on 2 October 2025 (Meeting No: 17; Decision No: 2025-17/06). Written informed consent was obtained from all participants, and the study was conducted in accordance with the principles of the Declaration of Helsinki.

### 2.7. Statistical Analysis

Data management and statistical testing were performed in SPSS version 25.0 for Windows (IBM Corp., Armonk, NY, USA). Distributional assumptions for numerical variables were examined through visual inspection of histograms and Q–Q plots, together with the Shapiro–Wilk test. Variables showing an approximately normal distribution are described using mean and standard deviation, whereas skewed data are summarized by median and interquartile range. Categorical findings are reported as counts and percentages.

Comparisons between two independent groups were made with Student’s *t*-test when parametric assumptions were satisfied and with the Mann–Whitney U test otherwise. Pearson’s chi-square test was used for categorical comparisons. The relationship between HALP and the eight-level ordinal retinopathy classification was assessed using Spearman’s rho.

Logistic regression was applied to two outcomes. In the first analysis, membership in the DR case cohort was modeled according to HALP, age, and sex. HALP was scaled in 10-point increments. Because documented diabetes status differed inherently between cases and controls, this analysis was regarded as exploratory and was not used to infer a retinopathy-specific effect. The second model was confined to participants with DR and examined the likelihood of PDR after accounting for HALP, age, sex, and HbA1c. Regression estimates are provided as odds ratios with 95% confidence intervals. Model calibration was assessed with the Hosmer–Lemeshow test.

The classification performance of HALP for distinguishing the case and control cohorts was evaluated by ROC analysis. The cut-off yielding the largest Youden J statistic was identified, and the corresponding sensitivity and specificity were calculated. All probability values were based on two-tailed testing, and values below 0.05 were accepted as statistically significant.

## 3. Results

A total of 209 participants were included, comprising 105 patients with diabetic retinopathy and 104 controls. As shown in Table 1, hemoglobin, albumin, HDL cholesterol, and HALP score were significantly lower in the diabetic retinopathy group, whereas age, sex distribution, platelet count, and lymphocyte count were comparable between groups.

Among patients with diabetic retinopathy, 51 (48.6%) had NPDR and 54 (51.4%) had PDR. Compared with the NPDR group, patients with PDR had a higher proportion of women, lower hemoglobin and albumin levels, higher HbA1c levels, and lower HALP scores (Table 2). Spearman correlation analysis further showed a weak but significant negative correlation between diabetic retinopathy stage and HALP score (rho = −0.260, *p* = 0.007).

In the exploratory logistic regression analysis, higher HALP score was associated with lower odds of belonging to the diabetic retinopathy case group in both the unadjusted and age- and sex-adjusted models. In the adjusted model, each 10-unit increase in HALP score was associated with a 15.0% reduction in the odds of case-group membership (Table 3).

Among patients with diabetic retinopathy, higher HALP score was associated with lower odds of PDR in the unadjusted model; however, this association was no longer statistically significant after adjustment for age, sex, and HbA1c. In the adjusted model, female sex and higher HbA1c levels remained independently associated with PDR (Table 4).

**Figure 1:**
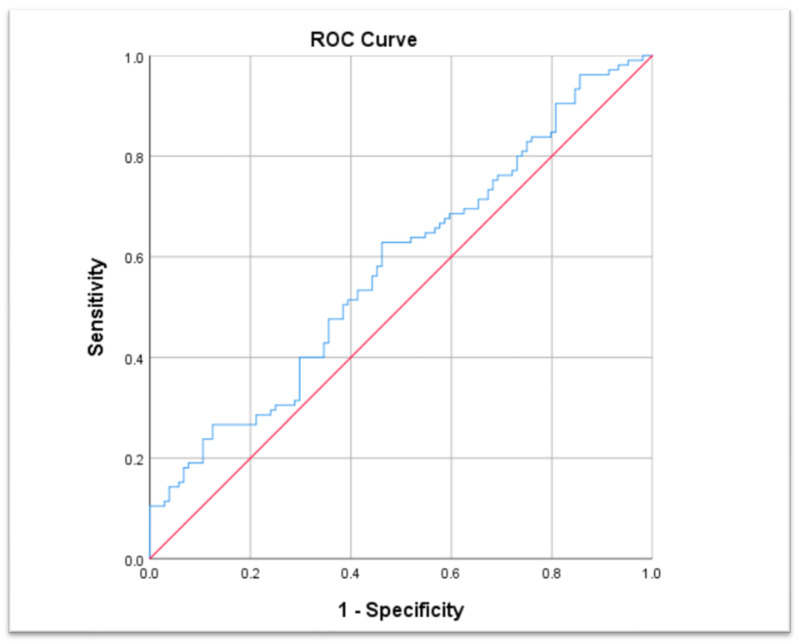
Receiver operating characteristic curve for HALP in classifying participants as diabetic retinopathy cases or controls. The blue line represents the ROC curve, whereas the red diagonal line represents the reference line corresponding to no discriminative ability (AUC = 0.500). The AUC was 0.579 (95% CI: 0.502–0.657; *p* = 0.048). The Youden-based threshold of ≤52.09 yielded a sensitivity of 62.9% and a specificity of 53.8%.

Receiver operating characteristic analysis demonstrated that HALP score had a statistically significant but weak ability to distinguish patients with diabetic retinopathy from controls (AUC = 0.579, 95% CI: 0.502–0.657, *p* = 0.048). At the optimal cut-off value of ≤52.09, sensitivity was 62.9% and specificity was 53.8% (Figure 1).

## 4. Discussion

The present study demonstrated that patients with diabetic retinopathy had significantly lower HALP scores than controls, and that higher HALP values were associated with lower odds of diabetic retinopathy case-group membership after adjustment for age and sex; however, this finding cannot be interpreted as a retinopathy-specific association because diabetes status differed intrinsically between the case and control groups. Among patients with retinopathy, HALP scores were lower in PDR than in NPDR, and HALP showed a weak but significant inverse correlation with ordered retinopathy stage. However, the association between HALP and PDR was attenuated after adjustment for age, sex, and HbA1c. These findings suggest that HALP may reflect systemic alterations accompanying diabetic retinal involvement, while its independent relationship with advanced retinopathy requires more cautious interpretation.

Our results are consistent with the limited but growing literature on HALP in diabetic retinopathy. Ding et al. reported an inverse, L-shaped association between HALP and DR risk in a nationally representative US diabetic population, while Wang et al. identified HALP as an independent correlate of DR in patients with type 2 diabetes [14,15]. A recent clinical study by Mutlu et al. also found lower HALP scores in patients with diabetic retinopathy and reported further reductions in proliferative disease [16]. The present study supports these observations in an ophthalmology-based sample and extends them by evaluating HALP not only in relation to DR presence, but also across clinically ordered disease severity. In the exploratory case–control model, each 10-unit increase in HALP score was associated with a 15% reduction in the odds of case-group membership. However, this association may reflect systemic differences related to diabetes as well as retinal involvement.

The lower HALP values observed in more advanced retinopathy are biologically plausible. HALP integrates hemoglobin, albumin, lymphocyte count, and platelet count, thereby reflecting, at least indirectly, oxygen-carrying capacity, nutritional–inflammatory status, immune balance, and platelet-related vascular activity [19,20,21]. In the current study, hemoglobin and albumin were both lower in patients with DR than in controls and were also lower in PDR than in NPDR. These findings align with prior population-based studies reporting inverse associations of hemoglobin and serum albumin with diabetic retinopathy [22,23]. Lower hemoglobin may intensify retinal hypoxic stress, whereas reduced albumin may reflect systemic inflammation, impaired antioxidant reserve, or broader vascular–metabolic dysfunction [22,23]. The fact that lymphocyte and platelet counts were not individually different between groups, while HALP was, further supports the value of examining integrated rather than isolated laboratory indicators in complex microvascular disease.

The PDR-specific regression findings provide an important boundary for interpretation. Although HALP was significantly associated with lower odds of PDR in unadjusted analysis, this relationship did not remain statistically significant after adjustment for age, sex, and HbA1c. In contrast, higher HbA1c remained independently associated with PDR, consistent with the central role of chronic glycemic exposure in progression to sight-threatening retinopathy. A recent Cochrane review concluded that increased HbA1c is likely associated with progression to PDR among individuals with diabetic retinopathy [24]. Accordingly, lower HALP may accompany more severe retinal disease, but its relationship with proliferative transformation may partly overlap with established metabolic drivers, particularly poor glycemic control.

The ROC analysis further clarifies the possible clinical role of HALP. Although HALP significantly discriminated patients with diabetic retinopathy from controls, the AUC of 0.579 indicates weak stand-alone diagnostic performance. This finding is broadly in line with prior work showing that HALP alone has limited discriminatory capacity for DR, whereas its performance may improve when incorporated into broader clinical models [15]. Therefore, HALP should not be viewed as a diagnostic substitute for ophthalmologic assessment, but rather as a low-cost, routinely available adjunctive marker that may contribute to systemic risk characterization in diabetic patients.

## 5. Limitations

Several limitations should be acknowledged. First, the single-center case–control design limits generalizability and precludes causal inference. Second, the control group consisted of ophthalmology outpatients without a documented diagnosis of diabetes rather than patients with diabetes but no retinopathy. Therefore, the observed difference in HALP score between the groups cannot be attributed exclusively to retinal involvement and may also reflect systemic alterations associated with diabetes itself. Glucose and HbA1c were not measured in the control group because study-specific glycemic testing was not performed in participants without a clinical indication; consequently, previously undiagnosed diabetes cannot be completely excluded. In addition, smoking status and alcohol consumption were not systematically recorded. Third, clinically relevant covariates such as diabetes duration, blood pressure, renal function, body mass index, medication use, smoking status, and comorbid inflammatory or nutritional conditions were not available for adjustment. Fourth, excluding patients who had previously undergone retinal laser photocoagulation, intravitreal injections, or vitreoretinal surgery may have introduced selection bias by underrepresenting previously treated and more advanced PDR cases, thereby limiting the representativeness of the PDR subgroup. Fifth, HALP was derived from a single laboratory measurement, preventing evaluation of longitudinal variability or predictive value over time. Finally, the subgroup sample may have limited statistical power to detect an independent association between HALP and PDR after multivariable adjustment.

## 6. Conclusions

In conclusion, lower HALP scores were observed in patients with diabetic retinopathy compared with ophthalmology controls without documented diabetes. However, the between-group association cannot be attributed exclusively to retinopathy because it may also reflect systemic alterations related to diabetes. Within the diabetic retinopathy group, lower HALP values accompanied more advanced disease in comparative and correlational analyses, but HALP was not independently associated with PDR after adjustment for age, sex, and HbA1c. Any potential role of HALP as an adjunctive biomarker therefore requires confirmation in appropriately designed longitudinal studies that include patients with diabetes without retinopathy as a comparator group.

## Figures and Tables

**Table 1:** Comparison of demographic and laboratory characteristics between patients with diabetic retinopathy and controls.

Variable	Diabetic Retinopathy Group (*n* = 105)	Control Group (*n* = 104)	*p* Value
Age, years	65 (14)	63 (14)	0.239 *
Female sex, *n* (%)	53 (50.5)	64 (61.5)	0.141
Hemoglobin, g/dL	13.62 ± 1.85	14.32 ± 1.40	0.002 **
Platelet count, ×10^9^/L	252.98 ± 68.90	255.19 ± 62.70	0.809 **
Lymphocyte count, ×10^9^/L	2.12 (0.94)	2.29 (1.01)	0.092 *
Albumin, g/L	41.60 (4.80)	41.90 (3.28)	0.012 *
HDL cholesterol, mg/dL	44.96 ± 10.65	50.03 ± 10.59	<0.001 **
HALP score	47.47 (26.44)	52.67 (31.83)	0.048 *

DR, diabetic retinopathy; HALP, hemoglobin–albumin–lymphocyte–platelet; HDL, high-density lipoprotein. * Mann–Whitney U test; ** independent-samples *t*-test. Sex distribution was compared using the chi-square test. Statistically significant *p* values are shown in bold.

**Table 2:** Comparison of demographic and laboratory characteristics between patients with non-proliferative and proliferative diabetic retinopathy.

Variable	NPDR Group (*n* = 51)	PDR Group (*n* = 54)	*p* Value
Age, years	63.00 (15.00)	67.50 (11.00)	0.106 *
Female sex, *n* (%)	16 (31.4)	37 (68.5)	**<0.001**
Hemoglobin, g/dL	14.28 ± 1.82	13.00 ± 1.67	<0.001 **
Platelet count, ×10^9^/L	246.61 ± 72.15	259.00 ± 65.80	0.360 **
Lymphocyte count, ×10^9^/L	2.14 (0.94)	2.11 (0.97)	0.651 *
Albumin, g/L	42.30 (4.80)	39.90 (5.28)	0.002 *
Glucose, mg/dL	149.00 (77.00)	177.00 (101.00)	0.286 *
HbA1c, %	7.40 (2.40)	9.10 (2.47)	<0.001 *
HDL cholesterol, mg/dL	44.20 ± 9.63	45.69 ± 11.58	0.477 **
HALP score	52.90 (42.49)	45.98 (17.62)	0.008 *

NPDR, non-proliferative diabetic retinopathy; PDR, proliferative diabetic retinopathy; HALP, hemoglobin–albumin–lymphocyte–platelet; HDL, high-density lipoprotein; HbA1c, hemoglobin A1c. * Mann–Whitney U test; ** independent-samples *t*-test. Sex distribution was compared using the chi-square test. Statistically significant *p* values are shown in bold.

**Table 3:** Exploratory logistic regression analysis of factors associated with diabetic retinopathy case-group membership.

Variable	Crude OR (95% CI)	*p* Value	Adjusted OR (95% CI)	*p* Value
HALP score, per 10-unit increase	0.870 (0.773–0.979)	**0.021**	0.850 (0.750–0.963)	**0.011**
Age, per 1-year increase	—	—	1.021 (0.989–1.054)	0.201
Female sex, vs. male	—	—	0.517 (0.287–0.928)	**0.027**

OR, odds ratio; CI, confidence interval; HALP, hemoglobin–albumin–lymphocyte–platelet score. Odds ratios for HALP represent the change associated with a 10-unit increase. Age was entered per additional year, and sex was coded as female versus male. The multivariable model included HALP, age, and sex. Model fit was satisfactory according to the Hosmer–Lemeshow test (*p* = 0.853). Statistically significant *p* values are shown in bold.

**Table 4:** Logistic regression analysis of factors associated with proliferative diabetic retinopathy among patients with diabetic retinopathy.

Variable	Crude OR (95% CI)	*p* Value	Adjusted OR (95% CI)	*p* Value
HALP score, per 10-unit increase	0.736 (0.600–0.901)	**0.003**	0.809 (0.640–1.023)	0.077
Age, per 1-year increase	—	—	1.021 (0.967–1.078)	0.455
Female sex, vs. male	—	—	3.892 (1.566–9.677)	**0.003**
HbA1c, per 1% increase	—	—	1.573 (1.169–2.117)	**0.003**

OR, odds ratio; CI, confidence interval; HALP, hemoglobin–albumin–lymphocyte–platelet score; PDR, proliferative diabetic retinopathy; HbA1c, hemoglobin A1c. The adjusted model included HALP score, age, sex, and HbA1c. HALP score was entered into the model per 10-unit increase. Statistically significant *p* values are shown in bold. Model calibration was acceptable according to the Hosmer–Lemeshow goodness-of-fit test (*p* = 0.199).

## Data Availability

The data presented in this study are available from the corresponding author upon reasonable request.
